# A bifractal nature of reticular patterns induced by oxygen plasma on polymer films

**DOI:** 10.1038/srep10126

**Published:** 2015-05-20

**Authors:** Junwan Bae, I. J. Lee

**Affiliations:** 1 Department of Physics, Research Institute of Physics and Chemistry, Chonbuk National University, Jeonju, 561-756, Korea

## Abstract

Plasma etching was demonstrated to be a promising tool for generating self-organized nano-patterns on various commercial films. Unfortunately, dynamic scaling approach toward fundamental understanding of the formation and growth of the plasma-induced nano-structure has not always been straightforward. The temporal evolution of self-aligned nano-patterns may often evolve with an additional scale-invariance, which leads to breakdown of the well-established dynamic scaling law. The concept of a bifractal interface is successfully applied to reticular patterns induced by oxygen plasma on the surface of polymer films. The reticular pattern, composed of nano-size self-aligned protuberances and underlying structure, develops two types of anomalous dynamic scaling characterized by super-roughening and intrinsic anomalous scaling, respectively. The diffusion and aggregation of short-cleaved chains under the plasma environment are responsible for the regular distribution of the nano-size protuberances. Remarkably, it is uncovered that the dynamic roughening of the underlying structure is governed by a relaxation mechanism described by the Edwards-Wilkinson universality class with a conservative noise. The evidence for the basic phase, characterized by the negative roughness and growth exponents, has been elusive since its first theoretical consideration more than two decades ago.

Dynamic scaling approach toward fundamental understanding of complex surface structures which often grow under far-from-equilibrium conditions holds technological significance in many areas including molecular beam epitaxy (MBE)^1^,^2^,^3^, chemical vapor deposition^4^,^5^,^6^, and plasma/ion-beam etching^7^,^8^,^9^,^10^,^11^. Considerable progress has been achieved in the fundamental understanding of fractal-like topographies of the various non-equilibrium surfaces, since the concept of dynamic scaling was introduced^12^. The dynamic scaling analysis allows one to define a universality class in which there are but a few essential factors determine the scaling exponents characterizing the scaling behavior. During the last several decades, a great deal of effort has been devoted to categorize various growth systems, displaying scale invariance in space and time, into a few universality classes through the examination of the spatial and temporal correlations of interface features. For a large body of a growing interface, it is known that the global interface width which characterizes the surface roughness for a system with a lateral size *L* scales according to the Family-Vicsek ansatz^12^,
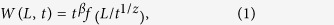
where the scaling function, *f*(*u*), behaves as
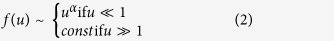


The roughness exponent, *α*, the dynamic exponent, *z*, and the growth exponent characterizing the short time behavior of the surface, *β*, define the universality class of a system under investigation. The scaling law of 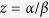, which links three global exponents, is valid for any growth process that obeys the scaling relation shown in Eq. (1).

Local growth dynamics have been widely described by the continuum Langevin equation given as
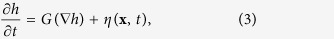
where 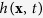 is the height of the surface at position **x** and time *t*. The noise term, 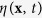 incorporating the random fluctuation in the deposition/etching process is usually considered to be uncorrelated in space and time, and is regarded as either nonconservative or conservative. The general function, 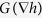, describing the relaxation mechanism is defined by the symmetries and dimensions of the growth dynamics under consideration. Therefore, the competition between the function, 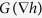, and the noise, 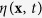, determines the universality class of an interfacial growth. Within the hydrodynamic limit 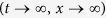, the simplest equation describing the fluctuation of an equilibrium interface is known as the Edwards-Wilkinson (EW) equation^13^,
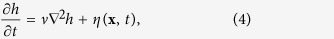
where the term 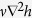 describes a smoothing effect driven by local chemical potential. The stochastic nature of the deposition flux generates a nonconservative noise satisfying 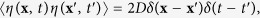 where 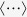 denotes an ensemble average and *D* is a constant. In the absence of the lateral growth described by 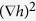 term, the seemingly oversimplified EW growth dynamics is known to control the asymptotic behavior of an ideal MBE growth equation^3^. Nevertheless, the realization of the EW equation is extremely rare in practice. So far, the sedimentation of silica nanospheres under gravitation was reported to belong to the EW universality class with characteristic exponents of 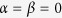 and *z* = 2 in (2 + 1)-dimensions^14^. The other type of randomness coming from the activated character of surface diffusion generates a conservative noise, which was considered two decades ago in combination with various types of conservative dynamics^3^,^15^. The diffusive noise with the property 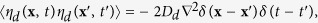 has long been thought to exist in real systems such as MBE, but no experimental evidence for the corresponding universality class has been discovered. In this report, we present the kinetic roughening of a plasma-etched polymer interface as the first experimental evidence for the EW universality class with a conservative noise. It is also demonstrated that bifractality assuming two sets of scaling exponents imbedded in the same physical space provides deeper insights into the formation and growth of complex nano-patterns on the surface of plasma-etched polymer films.

## Data and Discussions

**Figure 1:**
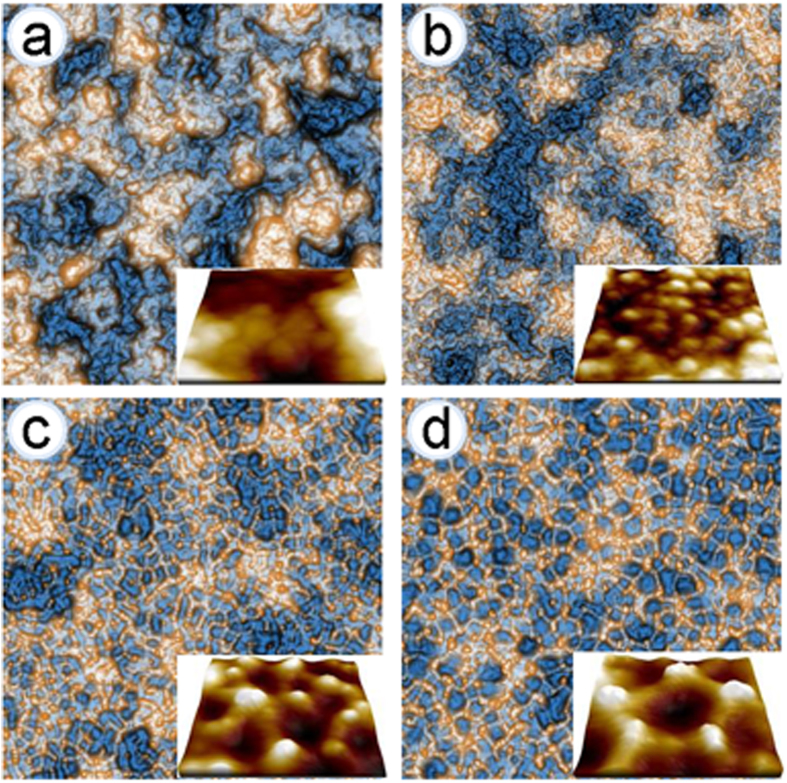
Topographic AFM images of parylene-C films obtained with scan sizes of 2×2 μm^2^ at various durations of O_2_-plasma exposure: (**a**) *t* = 0 s, (**b**) 60 s, (**c**) 360 s, and (**d**) 1,200 s. Perspective images cropped by dimensions of 250 × 250 nm^2^ are displayed in each corner.

**Figure 2:**
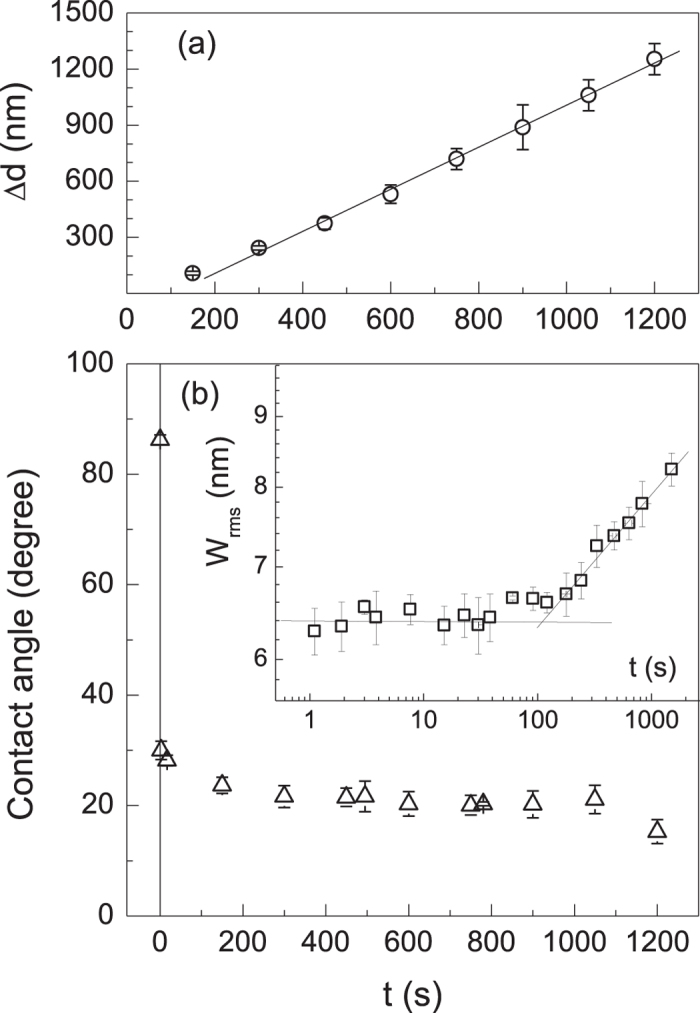
The etched film thickness (Δd) and the contact angle at various treatment times are shown in panel (**a**) and (**b**) respectively. Inset in the panel (**b**) displays the evolution of the rms roughness in log-log scale. The error bars show the standard deviation from the mean value determined from at least four different locations on the surface of each film.

The evolutions of the topographic atomic force microscopy (AFM) image of parylene-C films which are exposed to oxygen plasma for various durations are shown in Fig. 1. The surface becomes fully covered by small protuberances as the exposure time is increased above *t* = 10 s. The size of the protuberances and the separation between them (wavelength) grow with the duration of plasma exposure. Above *t* = 300 s, the protuberant spots bridged by low-lying structures start to form a fairly regular reticular pattern on the surface. As displayed in the insets, the underlying structures consisting of closely packed pits surrounded by polygonal sides grow with increasing exposure time above *t* = 300 s. The changes in the mechanical and chemical properties of the polymer surface due to the plasma exposure are shown in Fig. 2. A fairly uniform etch rate of 1.1 nm/s is observed during the plasma exposure above *t* = 300 s. While the root-mean-square (rms) roughness of the polymer surface determined by 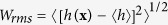 increases with a small power-law of 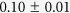, the static contact angles formed by a drop of 7 μL deionized water on the polymer film are drastically reduced to 20 degree and remain nearly the same above *t* = 300 s. In the light of previous study of high resolution X-ray photoelectron spectroscopy^16^, the contact angle data imply that the total surface concentration of polar functional group containing oxygen remains unchanged during the growth of the reticular pattern.

The local surface fluctuation at a fixed time can be described quantitatively by the height-difference correlation function (HDCF),
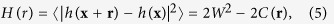
where 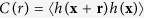 is the height-height correlation function (HHCF), *W* is the rms roughness or the interface width, and the average 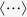 is calculated overall 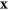 in windows of size 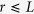. When anomalous scaling takes place, the correlation function scales as^17^,^18^,^19^
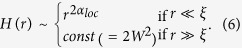


**Figure 3:**
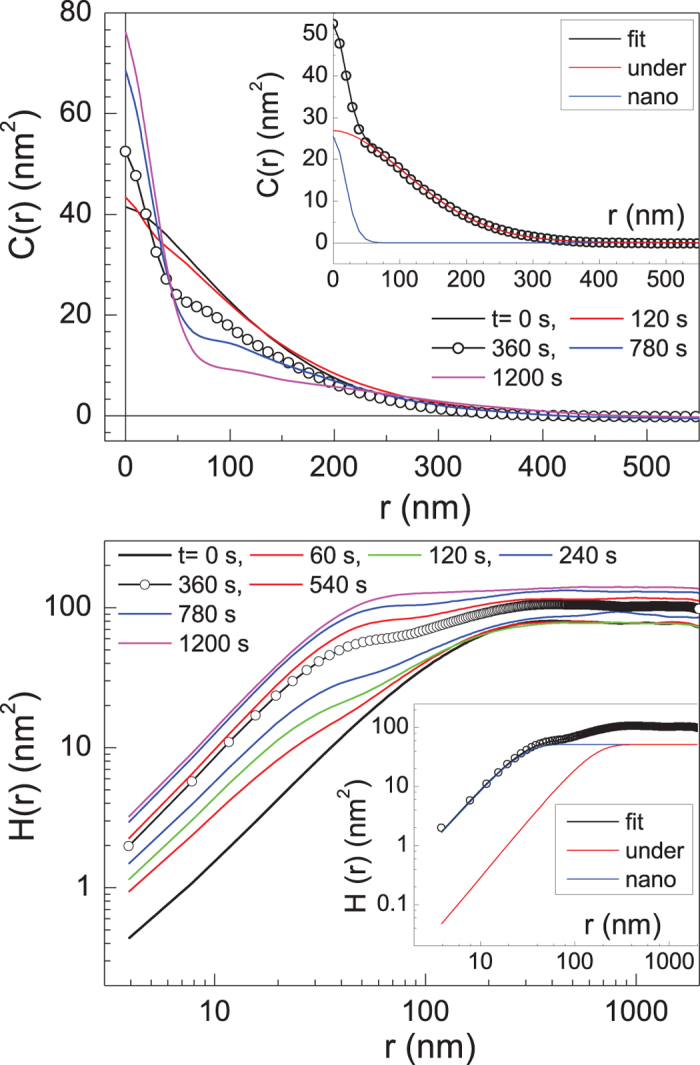
The top and bottom panel display 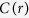 (HHCF) and 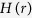 (HDCF), respectively, for the various treatment times. Inset in each panel shows a representative outcome of a bifractal interface fit for *t* = 360 s.

Since the correlation length *ξ* and the interface width *W* evolve over time as 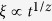 and 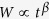, respectively, the standard self-affine Family-Vicsek scaling shown in Eq. (1) is recovered when the local roughness exponent, 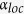, is equal to the global roughness exponent, 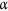. Figure 3 shows the radially averaged correlation functions of the polymer films treated for various durations. At *t* = 0 s, the power law dependence of $H(r)\sim r^{2\alpha_{\text{loc}}}$ in the small-*r* range becomes saturated at 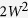
as the height fluctuations spaced a distance *r* apart lose their correlation when *r* ≈ *ξ*, which is exactly what is expected for the type of scaling shown in Eq. (1). The correlation functions are commonly modeled using the phenomenological scaling function, 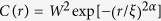, for a self-affine surface^20^. When the interface is exposed to oxygen plasma, an additional scaling behavior develops below 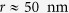 as the fairly regular distribution of protuberances grows in size. Taking into account the spatial and temporal progression of the correlation functions, it seems inevitable to consider the concept of a bifractal interface. Since the newly generated features are too small in real space to affect the underlying long wavelength features, we assume that the nano-size protuberances evolve statistically independent of the underlying structure. To extract *W* and 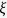 from each curve, we modeled a correlation function as the sum of two contributions, one from the nano-size protuberances and the other from the underlying structure as
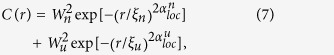
in which 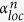
and 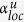
stand for the local roughness exponents for each contribution. Two insets of Fig. 3 show a representative outcome of the bifractal interface fit for the correlation functions 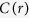 and 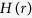 at *t* = 300 s. The curve marked as “nano” represents the nano-size protuberances and the other denoted as “under” stands for the underlying structure. As displayed, the large difference of the peak width (i.e., the correlation length, ξ) between two curves warrants very consistent fits. Each peak decreases falling close to Gaussian distributions characterized by the local roughness exponents, 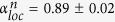 and 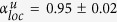 for the nano-size protuberances and the underlying structure, respectively. It is worth noticing from the inset of Fig. 3 that the height-difference correlation function of the bifractal interface satisfies the conventional scaling relation dominated by the nano-structures, namely $H(r)\sim r^{2\alpha_{\text{loc}}^{\text{n}}}$ for 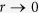.

**Figure 4:**
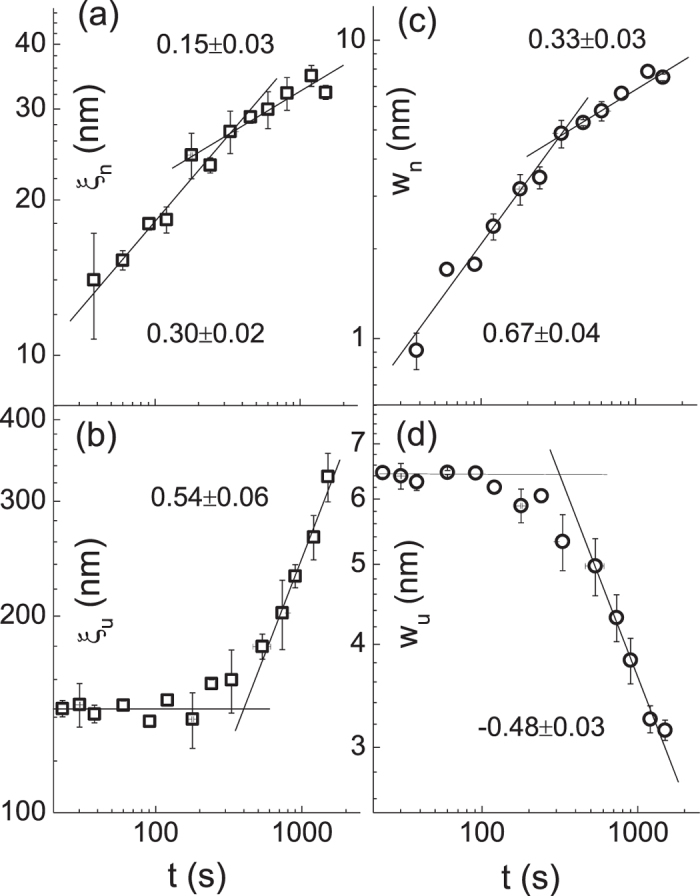
The temporal dependence of the correlation length, 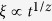, is shown in panel (**a**) and (**b**). The evolution of the interface width, 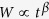, is displayed in panel (**c**) and (**d**). The subscripts *n* and *u* stand for the nano-size protuberances and underlying structure, respectively. The error bar on each data point indicates the standard deviation from the mean value determined from at least four different locations on the surface of each film. The measured slope and standard fitting error are written in each panel.

The results of bifractal analysis on the plasma-induced surface patterns are summarized in Fig. 4. The lines through the data points in each panel show a linear best fit of the scaling parameters in log-log scale. The crossover commonly occurring near *t* = 300 s is associated with the development of the low-lying bridges between the protuberant spots leading to the formation of the reticular pattern with closely packed pits. We notice that by the time at *t* = 330 s, the temperature of the polymer film under the oxygen plasma reaches the glass temperature known to take place at 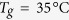
21. By applying the dynamic scaling law, 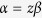, we obtain the global roughness exponent of 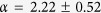 for the nano-size protuberances throughout the etching process, independent of the crossover. Interestingly, the height fluctuation of the underlying structure 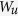 decreases over time with 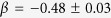, while the lateral size 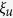 increases with 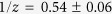, resulting in a negative roughness exponent of 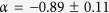 above *t* = 300 s. The transition from the layer-by-layer etching to deroughening process displayed in Fig. 4(d) is closely associated with the glass temperature of the polymer film. Nevertheless, the rms roughness given by 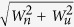 would actually increase with a positive slope of 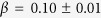 as shown in the inset of Fig. 2. In comparison with the data, we see that 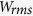 which was dominated by underlying structure in the early exposure times becomes controlled by the nano-size protuberances in the later times. Although the positive growth exponent (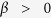) may agree with most of the kinetic roughening induced by either growth or etching process^22^, the dynamic scaling law based on a self-affine fractal would not work for the plasma-etched polymer surface.

**Figure 5:**
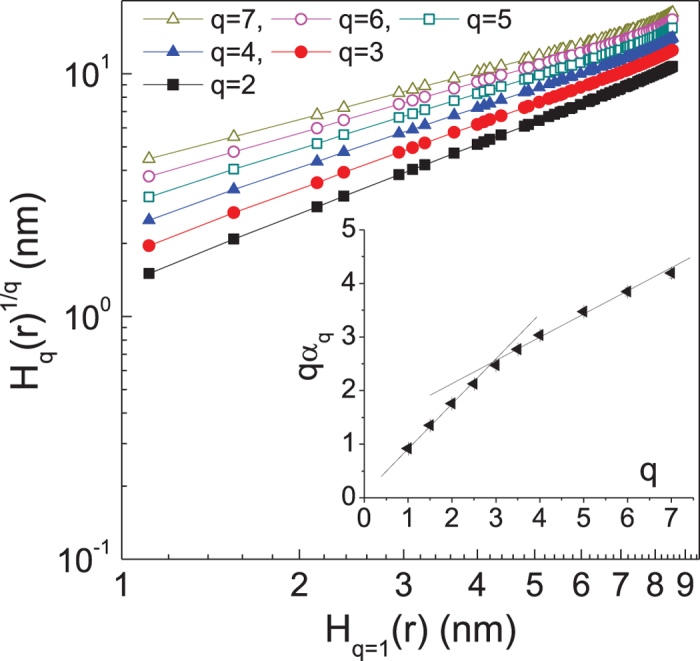
Log-log plot of 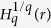 vs 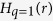 calculated from the AFM image displayed in Fig. 1(d). Inset displays the *q*th order scaling exponents 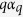 vs *q* for the film treated for *t* = 1,200 s. The size of error bar is smaller than the size of symbols. The lines are guides for the eyes.

Bifractality, a natural extension of self-affine fractal, assumes two scaling exponents 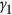 and 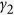 on fractal set of dimension D_1_ and *D*_2_, such that, as 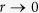, the local singularity of the interface is characterized by 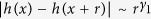 and 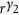. With the assumption, the *q*th-order height-difference correlation function 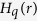 can be obtained as the superposition of two power-laws,
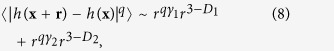
where the factor 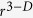 is the probability of being within a distance 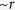 of the set of points having fractal dimension *D*. When 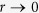, the power-law with the smallest exponent will dominate. Thus we obtain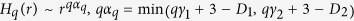. It indicates that, depending on the value of the exponent *q*, there exist two characteristic slopes (i.e., 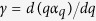) which act as local scaling exponents, 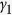 and 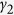. The *q*-dependent *q*th order scaling exponents displayed in inset of Fig. 5 support the bifractal behavior with a transition occurring near *q* = 3, which is qualitatively different from the multifractality observed in the native polymer films during the continuous growth regime^23^ in which 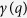 is obtained as a continuously varying function of the moment *q*. The plot of 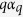 vs *q* is constructed using the scheme displayed in the main panel. The plot 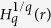 vs 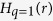, namely the extended self-similarity (ESS) plot^23^,^24^, significantly extends the scaling regime and provides an accurate estimate of the relative scaling exponents 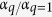. The bifractal nature of the *q*th order scaling exponents justifies the application of the correlation function with two different length scales as defined in Eq. (7).

**Figure 6:**
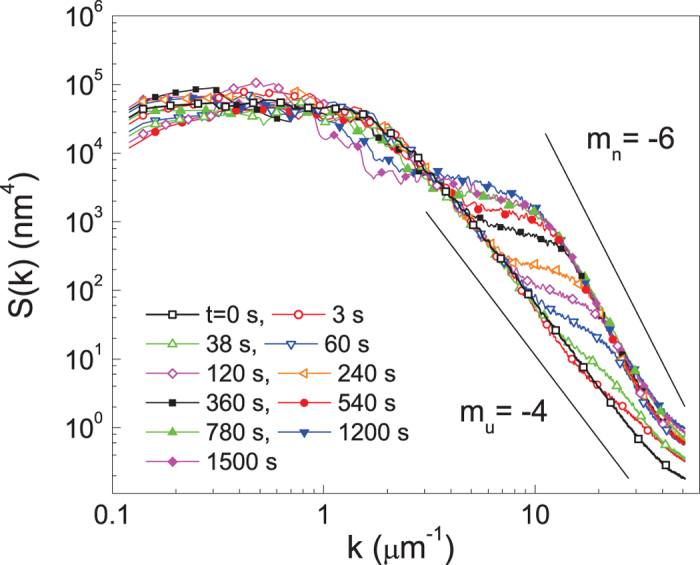
The power spectra obtained with various treatment times are displayed in log-log plot. The lines with negative slope are a guide for the eyes.

Complete characterization of the dynamic scaling of the growing surface requires examination of the power spectrum, 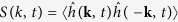, in which 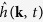 is the Fourier transform of the interface height. The dynamic scaling hypothesis shown in Eq. (1) is translated to the power spectrum in (2 + 1)-dimensions as^18^,^25^
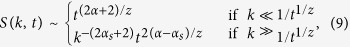
in which 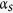 is the spectral roughness exponent. The power spectra of the polymer surface treated for various durations are shown in Fig. 6. For the native film (*t* = 0 s), the linear dependence (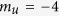) of the power spectrum in the regime of 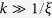 becomes saturated at 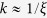, which is typical of Family-Vicsek type scaling expressed in Eq. (1). Under oxygen plasma, the polymer film develops an additional linear dependence 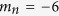 in the high-*k* region, as the small protuberances grow in size. Notice that, even with the growth of the nano-size protuberances, the underlying slope 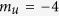 seems to be maintained. From the measured slope corresponding to the *k*-dependent exponent 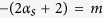, we find 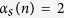 for the nano-size protuberances and 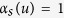 for the underlying structure. The temporal dependence of the power spectrum is described by two characteristic exponents 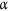 for the low-*k* regime and 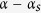 for the high-*k* regime. The absence of temporal dependence in the regime of 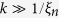 suggests that 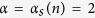, which is in good agreement with the result determined by the dynamic scaling law 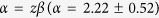 based on the data shown in Fig. 4. In the regime of 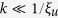, the absence of temporal dependence of the power spectrum suggests 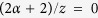, implying 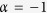 for the underlying structure, which is also consistent with the previous analysis based on Fig. 4 of 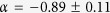. Unlike usual scaling theory in which originally flat interface becomes rough, the underlying structure evolves from the initially rough to a smooth interface until it reaches a small intrinsic roughness, independent of the system size. The roughness of the underlying structure is expected to decrease from initially rough interface following a power-law in time. The power-law dependence of the coalescing and deroughening of the underlying structure, i.e., 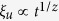 with 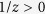 and 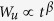 with 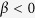, result in a negative roughness exponent. The unusual deroughening and concurrent coalescing mechanism displayed in the evolution of the underlying pits can be further analyzed in the context of generic scaling ansatz.

According to generic scaling ansatz^25^, the scaling relations 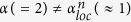 and 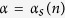 determined for the nano-size protuberances are consistent with super-roughening which occurs in conserved growth dynamics, such as diffusion driven by chemical potential gradient. For the underlying pit structure, we obtained the relations 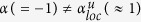 and 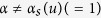, suggesting intrinsic anomalous scaling in which nonlocal effects such as shadowing and etchant redistribution effect^8^ may have been active during the etching process. However, when the value of 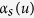 is strictly taken as a critical criterion^25^, the dynamic scaling of the underlying pits should belong to the border between super-roughening and intrinsic anomalous scaling. In fact, during the low power RF-plasma etching process, the typical nonlocal effects may not play a critical role in the restructuring process of the underlying pits above *t* = 300 s. As we discussed previously, the structural change occurring near *t* = 300 s is closely related to the glass temperature at 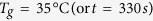. Moreover, it is interesting to notice that the growth dynamics of the underlying pits determined by the scaling exponents 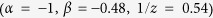 is consistent with the EW universality class with a conservative noise which is characterized by exponents of 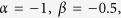 and 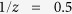 in (2 + 1)-dimensions^3^. The kinetic roughening of the underlying structure is described well within the most simple continuum growth equation. On the other hand, the characteristic scaling exponents of the nano-size protuberances, defined by Fig. 4(a), cannot be assigned to any known universality classes. Nevertheless, the kinetic roughening of the nano-structure at early times (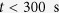) appears to resemble that of the initial growth of the polymer islands on a flat surface before the islands coalesce^26^, even though the detailed conditions for the surface growth are very different from each other.

Under oxygen plasma, the UV energy and oxygen species effectively break the polymer chains and terminate the chain-ends with the carbonyl group, resulting in low molecular weight materials, some of which are eventually desorbed under vacuum. Those remaining on the surface produce the nano-sized protuberances rich in polar molecules through diffusion and an aggregation process. The fairly regular distribution of the protuberances grows in size and wavelength, which is likely due to repulsive interactions between the protuberances under the plasma environment^16^. The initiation of the reticular pattern is closely related to the glass temperature of the polymer film occurring at 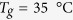. The results of bifractal analysis strongly suggest that the rubbery state above the glass temperature allows the underlying structure composed mainly of unbroken long chains to readily redistribute the irregularities on the surface, which is essentially the smoothing effect described by the EW term described by 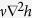. While the polymer films are etched away in the rate of 1.1 nm/s under the oxygen plasma, the generation and desorption of short polymer chains remain in equilibrium such that the total number of short chains on the surface is conserved, which seems compatible with conservative characteristics of the growth dynamics.

## Conclusions

The surfaces of plasma-treated parylene-C films have been thoroughly analyzed using various correlation functions. The characteristic scaling exponents are determined and verified independently. With the application of bifractal concept to the plasma-etched polymer surface, we are able to reveal otherwise impossible growth dynamics hidden underneath the nano-patterns, that is, the EW universality with a conservative noise which has not been realized since its first theoretical consideration more than two decades ago. The bifractal concept, a natural extension of the conventional dynamic scaling theory based on self-affine fractal, provides deeper insights into the formation and growth of complex nano-patterns induced by oxygen plasma on the surface of polymer films. The method should be applicable to other nano-patterns grown on various organic/inorganic surfaces in which the development of nano-structure is accompanied by the long wavelength background topography^9^,^10^,^11^.

## Experimental methods

Parylene-C (poly(chloro-*p*-xylylene)) films were deposited in a custom-built chemical vapor deposition reactor consisting of a sublimation furnace, a pyrolysis furnace, and a deposition chamber backed by a diffusion pump. Dimer molecules (di-chloro-di-*p*-xylylene) obtained from Daisan Kasei Co. (Japan) were sublimed in a quartz tube at 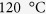 and then cracked into monomers in a pyrolysis furnace at 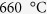. The monomers were subsequently condensed and polymerized on precut SiO_2_/Si substrates of 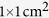 size in the deposition chamber, at room temperature. During deposition, the pressure was typically in the range of 1 mTorr, resulting in a growth rate of 20 nm/min. Several batches of polymer films, with approximately 40 samples of 1,700 nm think films in one batch, were grown with an identical condition. Plasma treatments were performed on several samples at the same time in an inductively coupled RF reactor for a predetermined duration. Once the reactor chamber reached the base pressure of 10 mTorr, the chamber was flooded with oxygen. The chamber pressure was regulated at 150 mTorr with a flow of oxygen before a power of 18 W was applied to the RF plasma coil in order to initiate the plasma etching. The sample temperature rose slowly up to 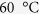 from room temperature after 25 min of plasma exposure. Changes in the film thickness before and after plasma treatment were accurately determined with spectroscopic ellipsometry. The average etch-rate was 1.1 nm/s. The effect of plasma treatment on the surface morphology was characterized by atomic force microscopy (AFM) using a non-contact mode with a pixel resolution of 512 × 512. Topographic AFM images were taken from at least four different locations on each polymer film with various scan sizes ranging from 2 × 2 to 5 × 5 μm^2^.

## Additional Information

**How to cite this article**: Bae, J. and Lee, I. J. A bifractal nature of reticular patterns induced by oxygen plasma on polymer films. *Sci. Rep.* **5**, 10126; doi: 10.1038/srep10126 (2015).
